# Melnick−Needles Syndrome Associated with Growth Hormone Deficiency: A Case Report

**DOI:** 10.4274/jcrpe.v1i5.248

**Published:** 2009-08-07

**Authors:** Leyla Akın, Erdal Adal, Mustafa Ali Akın, Selim Kurtoğlu

**Affiliations:** 1 Department of Pediatric Endocrinology, Erciyes University, Faculty of Medicine, Kayseri, Turkey; 2 Department of Pediatric Endocrinology, Bakırköy Maternity and Children’s Hospital, İstanbul, Turkey; 3 Department of Pediatrics, Erciyes University, Faculty of Medicine, Kayseri, Turkey; +90 352 437 49 37leylabakin@gmail.comDepartment of Pediatric Endocrinology, Erciyes University, Faculty of Medicine, Kayseri, Turkey

**Keywords:** Melnick−Needles syndrome, Growth hormone deficiency

## Abstract

Melnick−Needles syndrome is an X−linked dominant bone dysplasia characterized by a typical facies (exophthalmos, full cheeks, micrognathia, and malalignment of teeth), flaring of the metaphyses of long bones, s−like curvature of the lower extremities, irregular constriction in the ribs, and sclerosis of base of the skull. The phenotype of affected individuals varies, even within families. About fifty cases of Melnick−Needles syndrome have been reported to date. Short stature is not a well−known component of the disorder. There is only one reported case of Melnick−Needles syndrome associated with growth hormone deficiency.

A six−year−old girl who presented to our clinic with short stature was diagnosed as Melnick−Needles syndrome based upon characteristic clinical and radiological findings. Two different stimulation tests demonstrated growth hormone deficiency. Presenting this second case of Melnick−Needles syndrome associated with growth hormone deficiency, we suggest that this association may be coincidental, but that it may also be a consequence of craniofacial abnormalities or an independent component of the disorder.

**Conflict of interest:**None declared.

## INTRODUCTION

Melnick−Needles syndrome is an X−linked dominant bone dysplasia characterized by a typical facies and characteristic radiological findings. To date, about fifty cases of Melnick−Needles syndrome have been reported (1). The phenotype of affected individuals varies, even within families. Stature is known to be usually normal. Only one patient was reported to be with Melnick−Needles associated with growth hormone deficiency (2). In this report, a second patient is presented with Melnick−Needles syndrome with short stature and growth hormone deficiency.

## CASE REPORT

**Figure 1:**
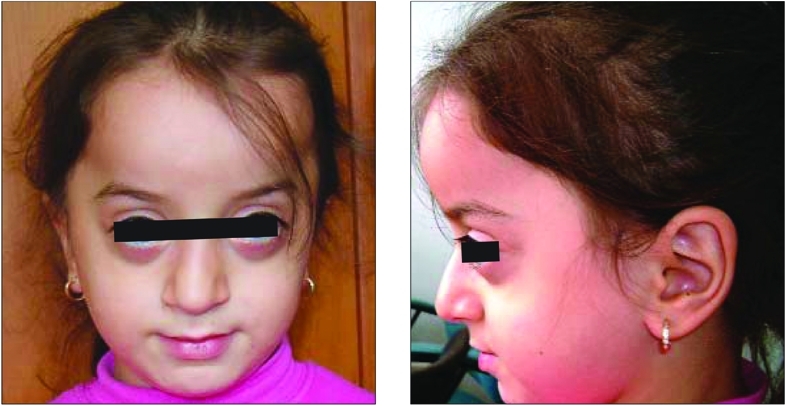
Characteristic facial appearance of the case

A six year−old girl was brought to our outpatient clinic for evaluation of her short stature. She was the second child of healthy nonconsanguinous parents. Physical examination revealed facial features typical for Melnick−Needles syndrome with exophthalmos, full cheeks, high forehead and micrognathia, malaligned teeth, mild genu valgum, narrow shoulders, and small chest wall with pectus carinatus (Figure 1). Her height was 103 cm (<3^rd ^percentile; SDS:−2.6) and her we ight was 21 kg (25^th^ percentile). She was prepubertal according to Tanner staging. She was reported to suffer from frequent severe upper respiratory tract infections. She had a hoarse voice. She was wearing glasses for myopia. Examination findings were otherwise normal. Family history was unremarkable for such a disorder. Her target height was 162 cm (0 SD). Laboratory results were as follows: Htc: 38%, Hb: 12 mg/dl, BUN: 10 mg/dl, creatinine: 1.29 mg/dl, Ca: 9.5 mg/dl (2.3 mmol/L), P: 4 mg/dl (1.2 mmol/L), ALP: 340U/L, AST: 35U/L, ALT: 28U/L. Thyroid function tests were normal. The patient’s bone age was evaluated as five years. Growth hormone stimulation tests using clonidine and L−dopa revealed peak responses of 7.3 μg/L and 9 μg/L, respectively, suggestive of partial growth hormone deficiency (normal≥10 μg/L). 

**Figure 2:**
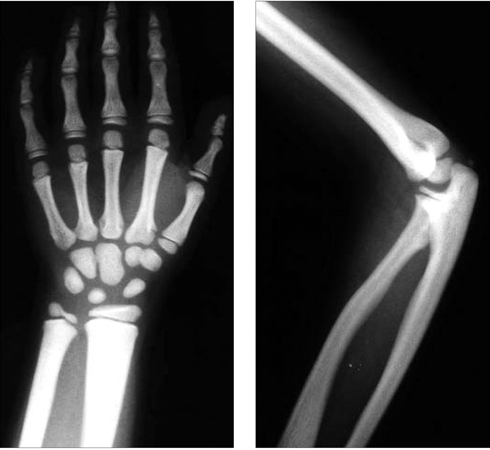
Characteristic facial appearance of the case

X−ray examination showed sclerotic appearance of the bones (Figure 2). Bowing of the radius, coxa valga, genu valgum, short distal phalanges with cone shaped epiphyses, a relatively small thoracic cage with irregular ribbon−like ribs and iliac flaring were also noted. Hypophyseal MRI was normal. 

The characteristic craniofacial and radiological findings led us to consider a diagnosis of Melnick−Needles syndrome. Ophthalmologic examination revealed no glaucoma. Cardiologic consultation did not disclose any cardiac abnormality. The patient was also investigated for any renal abnormality. Blood chemistry, urine analysis and ultrasonography were all negative for renal disease. X−rays of the mother showed no abnormality. It was not possible to conduct a genetic analysis to confirm the diagnosis.

## DISCUSSION

In 1996, Melnick and Needles described families who had several members with severe congenital bone disorder associated with typical facial features such as exophthalmos, full cheeks, micrognathia, malalignment of teeth, along with flaring of the metaphyses of the long bones, s−like curvature of the lower extremities, irregular constriction in the ribs, and sclerosis of the base of skull (3). Coste et al (4) had previously described a 58−year−old woman with a striking facies which included frog−like eyes, a high forehead, full cheeks, receding chin and had used the term “osteodysplasty” for the first time to describe the skeletal findings of this patient. These findings included a contracted pelvis, osteoarthritis of the lumbar spine, curved long bones, tortuous ribboned ribs, and deformed clavicles and scapulae.

Melnick−Needles syndrome has an X−linked dominant inheritance. Most of the reported cases are females. The disorder was initially assumed to have an autosomal dominant transmittance, but reexamination of male cases showed that they were normal at the time of investigation (5). Robertson et al (6) reported X−linked inheritance in 2003 and showed that the syndrome was caused by gain−of−function mutations in the gene encoding filamin A (FLNA; 30017). Besides, these researchers found that FLNA mutations were also responsible for oto−palato−digital syndrome, types 1 (OPS 1) and 2 (OPS 2), and frontometaphyseal dysplasia with overlapping clinical phenotypes. These syndromes are referred as the OPS−spectrum disorders. Unfortunately, the pathogenesis of the disorder has not yet been clarified. Swejcar et al (7) found an increased content of collagen which could explain the sclerosing bone process. Fryns et al (8) suggested that this condition was a generalized connective tissue disorder because of the hyperlaxity of the skin and joints. The absence of a family history indicates that our patient is also a sporadic case, like most of the reported cases. The disorder is usually lethal in males, especially in those born to affected mothers (9). Ter Haar et al (10) reported three male patients with Melnick−Needles syndrome associated with congenital glaucoma and heart defects. In 1995, Hamel et al (11) proposed the term “ter Haar syndrome”, distinct from Melnick−Needles syndrome, as a unique entity showing recessive inheritance. Wong et al (12) demonstrated the noncompaction of the left ventricle in a patient with Melnick−Needles syndrome. Our patient did not have any cardiac abnormality or glaucoma; thus, ter Haar syndrome was excluded. Severe mandibular hypoplasia in patients with Melnick−Needles syndrome can cause upper airway restriction and increased incidence of respiratory infections (13). The history of frequent upper respiratory tract infections in our patient might also be related to mandibular hypoplasia. Recently, Femiano et al (2) reported three members of the same family with Melnick−Needles syndrome, one of them being associated with growth hormone deficiency. In this presentation we report the second case which may be coincidental or may suggest that growth hormone deficiency is a component of the disorder. The response to growth hormone therapy is not known in patients with this bone disorder. Growth hormone therapy was not given to our patient because the parents did not give consent.

In conclusion, growth hormone deficiency should be taken into consideration in patients with Melnick−Needles syndrome, especially if associated with short stature.
